# Minerals and Cancer: Overview of the Possible Diagnostic Value

**DOI:** 10.3390/cancers14051256

**Published:** 2022-02-28

**Authors:** Sascha Venturelli, Christian Leischner, Thomas Helling, Olga Renner, Markus Burkard, Luigi Marongiu

**Affiliations:** 1 Department of Nutritional Biochemistry, Institute of Nutritional Sciences, University of Hohenheim, Garbenstraße 30, 70599 Stuttgart, Germany; sascha.venturelli@uni-hohenheim.de (S.V.); christian.leischner@uni-hohenheim.de (C.L.); thomas.helling@uni-hohenheim.de (T.H.); olga.renner@uni-hohenheim.de (O.R.); 2 Department of Vegetative and Clinical Physiology, Institute of Physiology, University of Tuebingen, Wilhelmstraße 56, 72074 Tuebingen, Germany

**Keywords:** cancer, biomarkers, minerals, copper, zinc, selenium, iron, iodine, phosphorus, calcium

## Abstract

**Simple Summary:**

Minerals are important but often overlooked compounds that are required for a variety of cellular biochemical processes and pathways that regulate cell proliferation. Their dietary imbalance, which is becoming more common in the diets of industrialized countries, is linked to an increased risk of cancer. The current review will present some of the most important minerals for human physiology and evaluate their potential application as cancer biomarkers.

**Abstract:**

Cancer is the second leading cause of death worldwide and is expected to increase by one-third over the next two decades, in parallel with the growing proportion of the elderly population. Treatment and control of cancer incidence is a global issue. Since there is no clear way to prevent or cure this deadly malignancy, diagnostic, predictive, and prognostic markers for oncological diseases are of great therapeutic value. Minerals and trace elements are important micronutrients for normal physiological function of the body. They are abundant in natural food sources and are regularly included in dietary supplements whereas highly processed industrial food often contains reduced or altered amounts of them. In modern society, the daily intake, storage pools, and homeostasis of these micronutrients are dependent on certain dietary habits and can be thrown out of balance by malignancies. The current work summarizes the data on minerals and trace elements associated with abnormal accumulation or depletion states in tumor patients and discusses their value as potential tumor-associated biomarkers that could be introduced into cancer therapy.

## 1. Introduction

After World War Two, the mean food intake increased drastically, reflected in an ongoing obesity epidemic [1,2]. On the other hand, the quality of the food did not. Specifically, there has been a decrease in several minerals in foods over the last five decades, most notably zinc, copper, and iron [3,4,5]. Such an occurrence is mirrored in the worldwide spread of “hidden hunger”, defined as a prolonged lack of vitamins or minerals (collectively referred to as micronutrients) intake [6,7]. For example, it has been estimated that the daily intake of selenium is half that of the reference nutrient intake [8].

Micronutrients are necessary for immunological functions as well as the general cellular metabolism [9]. For instance, it has been shown that deficiency in micronutrients, including minerals, worsens the pathogenesis of COVID-19 [10]. Moreover, given the link between an abnormal immune system and oncogenesis [11], identifying factors that affect the former can aid in predicting the latter. Quantification of biomolecules that can interfere with immune function, for example, could theoretically serve as an indicator for cancer risk. Micronutrients in general and minerals in particular are suitable for this purpose.

Given that the cellular biochemistry requires micronutrients, the ongoing and unnoticed subclinical deficiency of micronutrients might cause significant global health issues. In particular, the hidden hunger might increase the risk of cancer development. Consequently, the measurement of micronutrients in general and minerals in particular might help the early identification of people at higher cancer risk.

The present work aimed at addressing the potential of selected minerals as potential cancer biomarkers. This review will first highlight the major molecular mechanisms linking minerals to oncogenesis, followed by a report on clinical evidence relating mineral levels to cancer risk, with a focus on the most recent findings.

**Figure 1:**
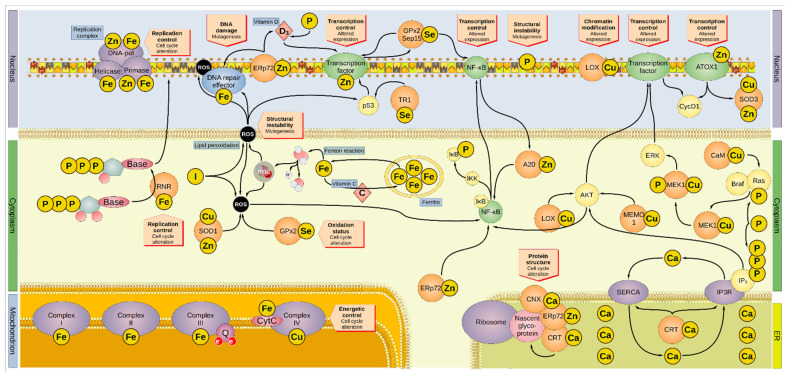
Overview of the involvement of minerals in oncogenesis. Minerals are involved in several and overlapping cellular pathways. Iron is sequestered by ferritin inside the cytoplasm. Iron leaking from the ferritin cages can react with water to form hydroxyl radical, one of the many ROS. Vitamin C promotes the reload of iron inside the ferritin cages. ROS can interact with lipid bilayers, generating more ROS molecules by lipid peroxidation, a process inhibited by iodide. In addition, ROS can directly damage the DNA and proteins, including the DNA-repair enzymes that are activated by ROS-induced damage. Furthermore, ROS activates p53 and NF-κB, altering the cell cycle. Iodide, SOD1, and GPx2 inactivate ROS. For instance, SOD1 removes superoxide radicals by dismutation reaction generating H_2_O_2_, O_2_, and GPx2 by producing two H_2_O from auto-reduction. The DNA-repair factors are modulated by the zinc-protein ERp72, which regulates the intake of vitamin D involved in the regulation of transcriptional factors. Cellular intake of vitamin D is also regulated by phosphorus. ERp72 reduces disulfide bonds in nascent proteins in the ER, in association with the calcium-proteins CNX and CRT. Moreover, ERp72 regulates NF-κB, the latter being also modulated by GPx2 and Sep15. GPx2 and Sep15 promote their own expression. A20 and AKT also activate NF-κB, which then reduces the activity of A20 in a negative feedback loop. AKT is modulated by LOX, MEMO1, and IP3, and affects the cell cycle by promoting the expression of CycD1. LOX can alter genetic expression by modifying the histones. Some transcription factors contain zinc, and a transcription regulator is p53, which TR1 modulates. ATOX1 is a zinc-containing protein that can modulate gene expression, particularly that of cyclin D1 and SOD3 (which regulates the oxidative environment outside the cell). Genetic transcription and cell cycle are regulated by ERK, which MEK1 activates after being phosphorylated by the complex Ras/Braf. One of the modulators of Ras is CaM, but also phosphorus can directly boost its activation. Similarly, phosphorus is also part of active IP3 that, aside from its direct modulation of AKT, regulates the release of calcium (effectively a second messenger on its own right) from the ER via the Ca^2+^ channel IP3R. The P-type ATPase SERCA mediates the transport of cytosolic calcium back into the ER. CRT regulates both IP3R and SERCA. Moreover, minerals are involved in DNA replication since they are embedded in several subunits of the DNA replication complex (namely: iron in the helicase, primase, and DNA-polymerase α, the latter two also containing zinc). Iron is present in the first three mitochondrial complexes and CytC, whereas copper is present in complex IV. Thus, minerals are essential to the energetic balance of the cell and its oxidative state. AKT, Ak strain transforming; A20, zinc finger protein A20; ATOX1, antioxidant-1; Braf, rapidly accelerated fibrosarcoma isoform B; CaM, calmodulin; CNX, calnexin; CRT, calreticulin; CycD1, cyclin D1; CytC, cytochrome c; DNA-pol, DNA polymerase; ER, endoplasmic reticulum; Erp72, endoplasmic reticulum resident protein 72; ERK, extracellular signal-regulated kinase; GPx2, glutathione peroxidase 2; IκB, NF-κB inhibitor; IKK, IκB kinase; IP3, inositol 1,4,5-trisphosphate; IP3R, inositol trisphosphate receptor; LOX, lysyl oxidase; MAPK, mitogen-activated protein kinase; MEK1, MAPK/ERK kinase1; MEMO1, mediator of cell motility 1; NF-κB, nuclear factor kappa-light-chain-enhancer of activated B cells; Q, coenzyme Q; Ras, rat sarcoma virus; RNR, ribonucleotide reductase; ROS, reactive oxygen species; Sep15, selenoprotein of 15-kDa; SERCA, sarco-/endoplasmic reticulum calcium ATPase; SOD, superoxide dismutase; TR1, thioredoxin reductase 1.

The recommended daily intake and associated serum levels of the most common minerals are summarized in Table 1 and serve as the basis for the categorization of these in the research cited here. Hence, this review focuses on the possible role of minerals as diagnostic markers for cancer providing an overview of the different molecular pathways that link these micronutrients to the oncogenic process. Figure 1 summarizes the findings of the present work.

## 2. Copper and Zinc

Copper plays a fundamental role in the oxidation reactions; thus, it is part of many enzymes like mono- and dioxygenases, dehydrogenases, oxidases, and reductases [22]. Cu transporter 1 (CTR1) transports dietary copper as Cu^+^ into epithelial cells of the small intestine, from where it is then transferred by ATPase copper transporter 7A (ATP7A) to the portal vein and finally released into the bloodstream from liver hepatocytes via ATP7B [23]. Genetic mutations affecting ATP7A result in Menkes disease (MD), a congenital condition characterized by the enterocytes’ inability to release copper in the portal vein and generalized copper deficiency. Children affected by MD have limited life expectancy and suffer from growth retardation, osteoporosis, and cellular fibers defects [24]. In the blood, most of the copper is carried in the serum bound to ceruloplasmin and in minor amounts associated with albumin and transcuprein. Hepatocytes accumulate copper, which is subsequently either transported to other cells through the bloodstream or expelled in the bile. Copper is uptaken by the CTR1 on the surface of the cells and stored associated with metallothioneins [25]. Inhibition of the CTR1-copper axis was recently described to diminish AKT signaling and therefore tumorigenesis [26]. Since several studies have reported increased levels of serum or tissue copper in cancer patients than healthy controls, copper has gained interest for therapeutic purposes [27]. Chelators that lower cellular copper bioavailability or ionophores that increase cellular copper bioavailability, respectively, are promising compounds for cancer treatment [23]. Its potential as a drug candidate for cancer therapy when present in a suitable complex was hypothesized to be due to formation free radicals. However, the unfavorable solubility in physiological buffer systems and the sometimes difficult-to-predict mechanism of action pose problems [28]. The concentrations of copper and zinc are tightly bound, and an increased Cu/Zn ratio has been observed in a plethora of cancer types [29,30,31,32,33,34]. Copper cellular concentration is regulated, among others, by mouse U2af1-rs1 region 1 protein (MURR1), which is targeted for ubiquitination by X-linked inhibitor of apoptosis (XIAP) [35,36].

There are about 50 human proteins containing copper, the majority being membrane transporters, that play a major role in metastasis due to their involvement in angiogenesis and cell-to-cell interactions [37,38,39]. Some of the non-transporter copper-proteins alter the cell cycle at critical points. Lysyl oxidase (LOX), as well as the secreted protein acidic and rich in cysteine (SPARC), mediate the cross-linking of the extra-cellular matrix, actin polymerization, and the activation of the phosphoinositide 3-kinases (PI3K) signaling pathway, thus promoting cellular proliferation and mobility [40,41]. The mediator of cell motility 1 (MEMO1) is also an activator of the PI3K signaling pathway [42]. In addition, LOX might be involved in chromatin remodeling by chemically modifying histones [43]. The mitogen-activated protein kinase kinase 1 (MAPKK, also known as MEK1 (MAPK/ERK kinase 1). or MAP2K1) activates the mitogen-activated protein-kinase (MAPK) signaling pathway, which is involved in proliferation and metastasis [44,45,46,47]. Antioxidant protein 1 (ATOX1) is a transcription factor that increases the expression of cyclin D1 and the extra-cellular super-oxide dismutase isoform 3 (SOD3) [48,49,50].

Copper also influences autophagy, a cellular process where dysfunctional biological components generated by reactive oxygen species (ROS) activity are sequestered inside specific vesicles (autophagosomes) to maintain homeostasis [51]. Removing damaged biological molecules from the cellular environment protects the cells from apoptosis with the consequence of effectively extending tumor survival [52]. Copper in particular increases the activity of the Unc-51-like autophagy activating kinase (ULK) complexes 1 and 2, which are involved in autophagosome formation and thus promote cancer proliferation [53,54].

Zinc is structurally embedded into over 300 proteins located in the cytoplasm, mitochondria, Golgi apparatus, and nucleus (e.g., in metalloproteases, histone deacetylases, dehydrogenases, hydrolases, transcription factors, DNA polymerase α, primase, etc.) involved not only in gene expression but also in the oxidative status of the cell, thus, their disruption is strongly linked to oncogenesis [55,56,57]. The association between zinc and cancer is not completely understood, but it is known that this micronutrient is associated to both immune response and cellular proliferation. In particular, zinc is involved in the regulation of several actors of the cell signaling pathways including protein kinase C, cAMP-dependent protein kinase A, phophodiesterase, and nuclear factor kappa-light-chain-enhancer of activated B cells (NF-κB) [58]. NF-κB is part of the Wingless and Int-1 (Wnt) signal pathway, and the targets of this transcription factor include around 200 genes involved in cell proliferation, metastasis, immune response, and inflammation [59,60]. A20 is a zinc-finger NF-κB suppressor whose expression is controlled by NF-κB, establishing a negative feedback loop for the Wnt signaling pathway, and it has been described as having both promoting and inhibiting carcinogenic properties [61]. Alteration of zinc levels, therefore, can have substantial consequences in cellular proliferation, inflammatory state, and immune response [62].

Zinc is also contained, together with copper, within the enzymes SOD1 and SOD3, which neutralize ROS [63]. SOD1 is prevalently recovered in the cytoplasm but it is also present in the nucleus and other cellular compartments; SOD3, instead, is present on the extracellular environment [64]. The super-oxide anion (O2•^−^), which belongs to ROS, is produced during the biochemical reduction of molecular oxygen, for instance in the mitochondrial electron transport chain (O_2_ + e^−^ → O_2_•^−^). ROS can cause damage to virtually all biological species within the cell, including DNA strands, proteins (inclusive of the enzymes involved in the DNA damage repair and DNA replication), and lipidic membranes [65]. The super-oxide anion can react with nitric oxide (NO), which has anti-inflammatory properties, to form peroxynitrite (O_2_•^−^ + NO• → ONOO^−^) that can damage cellular biomolecules, for instance causing lipid peroxidation, and trigger inflammation [66]. While zinc has a structural function, copper is part of SOD’s catalytic site and is involved in the reaction of neutralization of super-oxide anion: 2O_2_•^−^ + 2H^+^ + Cu^2+^ → H_2_O_2_ + O_2_ + Cu^+^ [64]. Unsurprisingly, alteration in SOD’s activity is associated with a wide range of diseases [67].

Furthermore, depletion of dietary zinc has been shown to alter the gut microbiota in terms of species richness and diversity, as well as genetic expression [68]. Since the alteration of the gut microbiota composition is associated with an increased risk of carcinogenesis [69], zinc levels might pinpoint cancer risk.

Analysis of 26 breast cancer tissues by mass spectrometry reported a significantly higher concentration of zinc in tumoral mass (3.5–19.5 parts per million (ppm)) than in the surrounding stroma (0.8–11.4 ppm), with a constant ratio of stroma over cancer zinc levels of 2.9 ± 1.6 [70]. A comparison of 27 oral squamous cell carcinoma patients and 86 controls reported a non-significantly increased zinc intake in the former group (12,851 mg daily) than in the latter (11,788 mg daily) group (Mann–Whitney U test *p*-value = 0.136) [71].

A survey of 989 hepatocellular carcinoma (HCC) patients did not show an association between zinc concentration and liver cancer, but copper and the ratio of copper over zinc did. When comparing patients in the upper against the lowest quartiles, the hazard ratio (HR) for overall survival (OS) to cancer was 2.06 (1.36–3.11, test for trends *p*-value < 0.01) for copper alone and 1.43 (0.99–2.08, test for trends *p*-value = 0.01) for the Cu/Zn ratio [72]. Blood zinc levels in patients with squamous cell carcinoma of the oral cavity were reduced by approximately half (*t*-test, *p*-value < 0.001) compared to healthy controls [73]. Zinc and copper were measured in colorectal cancer (966 cases and 966 matched controls) [29]. High zinc concentration was associated to reduced cancer risk (odds ratio (OR) = 0.65, 95% confidence interval (CI): 0.43–0.97, test for trends *p*-value = 0.07), whereas copper had the inverse trend (OR = 1.50, 95% CI: 1.06–2.13, test for trends *p*-value = 0.02). The ratio of copper over zinc was also associated with higher cancer risk (OR = 1.70, 95% CI: 1.20–2.40, test for trends *p*-value < 0.001).

The mean concentration of copper in pancreatic cancer patients (*n* = 100) was 1432 µg/L compared to the 1098 µg/L observed in the matched control group (*n* = 100), and the comparison of people with copper levels in the highest two quartiles and the lowest indicated a higher risk of cancer in high concentration of this micronutrient (OR = 4.9, *p*-value < 0.001) [74]. In particular, the threshold of 1215 µg/L was identified as the level above which cancer increased drastically.

Blood copper levels in patients with squamous cell carcinoma were about 45.5% higher (*t*-test, *p*-value < 0.001) compared to healthy controls [73]. Spectroscopic analysis of brain sections reported a significantly higher copper concentration in tumoral masses than in surrounding healthy tissues (0.0079 and 0.037 µg/cm^2^, respectively; Mann–Whitney U test *p*-value < 0.05) [75]. Conversely, in the same sections, zinc had higher levels in tumoral masses than healthy tissues (0.0403 and 0.0285 µg/cm^2^, respectively; Mann–Whitney U test *p*-value < 0.05). Others have measured 3.9–9.1 times lower copper levels and 2.4–9.6 times lower zinc levels in the tumoral mass than in the surrounding brain tissues [76]. Copper levels in brain tumor patients were significantly higher than in healthy controls (*t*-test *p*-value < 0.001) [77].

Copper and zinc were suggested as prognostic markers. A cohort of 175 HCC patients reported that serum levels above 68.3 μg/dL and 81.1 μg/dL for copper and zinc, respectively, allowed to identify patients at increased mortality risk with a sensitivity 78.3% and 60.4%, and a specificity of 48.1% and 65.2% [78]. Combining these two micronutrients as Cu/Zn ratio gave a sensitivity of 68.1% and a specificity 75.5% for a cut-off set at 0.999.

## 3. Selenium

Until 1957, selenium was considered a toxin, but studies on liver necrosis in rats revealed the biochemical importance of this element [79]. The effect of selenium on cellular biochemistry is proportional to its concentration. Selenium is an antioxidant at nutritional doses, but an oxidant at high (typically pharmacological) doses [80]. While the latter is used to treat tumors because cancerous cells are more susceptible to the oxidative action of selenium [81], dietary selenium deficiency is expected to rise in the near future [82]. The primary natural source of selenium, chiefly in the inorganic forms of selenate (SeO_4_^2–^) and selenite (SeO_3_^2−^), is diet (notably fish and meat), whereas non-natural sources of selenium include air pollution and cigarette smoke [83]. Deficiency in selenium intake can result in cardiomyopathy, degenerative disorders (including Alzheimer’s disease), and immunological dysfunctions, whereas chronic exposure to high levels of this element (selenosis) may lead to hair loss, skin rash, fatigue, and irritability [84,85].

The organic form of selenium is principally selenocysteine (Sec) which is incorporated into 25 human genes [86]. The triplet UGA, when present in the context of stem-loop structure known as Sec insertion sequence (SECIS), is recognized by a tRNA initially aminoacylated with serine and later converted to Sec [87]. Selenoproteins are widespread in all living kingdoms, including viruses, and all are oxidoreductases with Sec as the catalytic residue [88]. Thioredoxin reductase 1 (TR1), selenoprotein of 15 kDa (Sep15), and glutathione peroxidase 2 (GPx2) are the most studied members of this family in relation to cancer [89]. The main function of TR1 is maintaining the protein structural stability by keeping exposed cysteine residues in reduced form [90], but TR1 can also activate the tumor suppressor p53 [91]. Its overexpression in cancer tissues and cell lines suggests its involvement in cancer promotion [92]. Sep15 is also involved in maintaining the structural integrity of several proteins [93]. Like TR1, Sep15 is also overexpressed in several cancer types [94] and, although its oncogenic involvement is not clear, it has been suggested that it might be involved in cell cycle regulation and interferon-γ mediated inflammation [95]. GPx2 is an antioxidant [89], thus, its imbalance can affect the cellular environment at several levels. In addition, GPx2 is regulated by the Wnt pathway [96]. The link between selenium and DNA damage fostered a widespread interest in the association between selenium concentration and risk of cancer and the administration of selenium as an anti-cancer treatment [97].

The levels of selenium were not linked to the development of any type of cancer [98], whereas other studies reported that the median blood selenium was 58.8 µg/L in carcinoma patients compared to 84.7 µg/L of healthy controls [99]. Serum selenium in lung cancer patients (*n* = 48) was higher than in healthy controls (*n* = 39), with values of 166.0 and 144.7 ng per gram of serum, respectively, but the difference was not statistically significant [100]. A comparison of patients with serum selenium in the upper tertile (77.8 µg/L) to those in the lower tertile (50.8 µg/L) in a group of 302 patients results in a higher risk of lung cancer (HR = 1.64, 95% CI: 1.14–2.37, test for trends *p*-value < 0.01) [101]. Conversely, serum selenium was lower in renal cancer patients (161.7 µg/L, *n* = 104) than in healthy controls (228.8 µg/L, *n* = 774), resulting in an OR of 0.14 (95% CI: 0.10–0.20, test for trends *p*-value < 0.01) [102].

The amount of selenium in prostate tissues was not significantly different (*p*-value = 0.347) between cancer patients (*n* = 49) and healthy controls (*n* = 49), with mean concentrations of 191 and 168 µg/kg, respectively [103]. A comparison of prostate cancer cases (*n* = 467) and controls (*n* = 936) showed no statistical association between serum levels of selenium [104]. However, the stratification by ethnicity showed a trend (*p*-value = 0.02) for African-Americans, with the OR of the third over the first tertile being 0.59. Selenium serum levels were, instead, significantly lower in hepatoma patients (*n* = 187, mean concentration: 67.47 μg/L) than in healthy controls (*n* = 120, mean concentration: 108.38 μg/L) [105]. A study of 966 colorectal cancer cases and 966 matched healthy controls reported no significant differences in serum selenium levels between the two groups (84.0 and 85.6 µg/L, respectively), but stratification by gender showed a statistically significant decrease in cancer risk in women with the higher concentration of selenium than women with the lowest concentration (incidence rate ratio = 0.83, 95% CI: 0.70–0.97, test for trends *p*-value 0.032) [106]. Higher selenium was reported to be significantly higher (univariate ANOVA *p*-value < 0.001) in esophageal tumors than in healthy surrounding tissues [107].

The mean concentration of selenium in pancreatic cancer patients (*n* = 100) was 60 µg/L compared to the 76 µg/L observed in the matched control group (*n* = 100), and the ratio of people with selenium levels in the lower two quartiles and the upper indicated a higher risk of cancer in the presence of depletion of this micronutrient (OR = 41, *p*-value < 0.001) [74]. In particular, the threshold of 67 µg/L was identified as the level below which cancer increased drastically. In liver cancer patients, serum selenium (52.5 µg/L) was lower than the average level of healthy controls [108]. Specifically, 93.7% of the patients had serum levels below the threshold of 70 µg/L indicating dietary deficiency for this micronutrient.

Similarly, in breast cancer, women in the upper quartile of serum selenium concentration had a lower risk of mortality than women in the lowest quartile (HR = 0.63, 95% CI: 0.44–0.89) [109]. Blood selenium levels dropped from 74.3 µg/L in breast cancer patients with tumors smaller than 2 cm in diameter to 64.8 µg/L in those with tumors larger than 5 cm in diameter (χ^2^ *p*-value = 0.03) [110]. In the same study, the ratio of breast cancer patients with serum selenium in the first and fourth quartiles was associated to a higher mortality rate (HR = 2.03, 95% CI: 1.12–3.65, *p*-value = 0.02). Conversely, the comparison of 27 oral squamous cell carcinoma patients and 86 controls reported higher selenium intake in the former (142.9 µg daily) than in the latter (106.7 µg daily) group (Mann–Whitney U test *p*-value = 0.002) [71].

## 4. Phosphorus

Phosphorus is one of the most abundant elements in the human body (after oxygen, hydrogen, carbon, nitrogen, and calcium), accounting for about 1% of body weight and being stored in the bones as hydroxyapatite (Ca_10_(PO_4_)_6_(OH)_2_) [111]. Dietary phosphorus is usually present in organic form (embedded mostly in proteins, nucleic acids, and phospholipids) and requires specific enzymes to be recovered during digestion, hence its adsorption rate has relatively low efficiency [112]. Phosphorus is present in virtually all types of food [113], thus, even if deficiency of this mineral has been described and was associated with a series of diseases, chiefly bone demineralization and myopathy, it is hyperphosphatemia that is relevant in developed countries [114]. In particular, inorganic phosphorus, in the form of phosphoric acid (H_3_PO_4_) is a common additive in soft drinks and foodstuff and it is rapidly absorbed with approximately 100% efficiency [112,115,116]. The addition of phosphates to processed foods and canned drinks almost doubles the daily intake of phosphorus [112,117,118]. Excess dietary phosphorus is linked to arteriosclerosis, renal dysfunction, premature aging, and cancer [119]. Intake of phosphorus is complicated by the fact that it is entangled with that of calcium and vitamin D, whose imbalance is also associated with an increased risk of cancer [120]. Moreover, even dietary fructose acts as an additional bias because it decreases the intestinal adsorption of phosphate [121].

Vitamin D, in the active form of calcitriol, increases intestinal phosphate absorption while decreasing renal excretion, thereby increasing phosphorus serum levels, whereas hyperphosphatemia decreases calcitriol serum levels [122]. Calcium, on the other hand, chelates phosphates, thus reducing the serum levels of phosphorus [123]. The interrelation between calcitriol and phosphate generates a feedback mechanism that maintains the homeostasis of phosphorus in the human body, but chronic hyperphosphatemia determines the obliteration of the protective role of vitamin D, increasing the risk of cancer [123]. Vitamin D could reduce the insurgence of dysbiosis, reducing the risk of developing colon cancer [124]. Interestingly, patients of different cancer types showed consistent hyperphosphatemia [125] and cancer cells derived from several tissue types showed high cellular levels of phosphate [126].

Experiments on mice showed that high phosphorus intake increases the risk of lung cancer by activating the PI3K signaling pathway [127], whereas studies on cell lines showed that phosphate can directly activate the protein kinase B (Ak strain transforming (AKT)) [128]. The aberrant activation of the inositol 1,4,5-trisphosphate (IP3)/AKT signaling pathway due to excess phosphate in the cells can explain the association of hyperphosphatemia with several types of cancer [117]. Another role associated with high increase in phosphorus intake is the fact that phosphate makes insoluble complexes with calcium, depleting this mineral from binding bile acids known to foster bacteriosis and oncogenesis [129,130,131].

Spectroscopic analysis of brain sections reported a significantly lower phosphorus concentration in tumoral masses than in surrounding healthy tissues (1.71 and 3.01 µg/cm^2^, respectively; Mann–Whitney U test *p*-value < 0.05) [75].

Comparison of men (*n* = 4123) in the upper over the lower quintile of the phosphorus dietary intake showed a non-significant risk of prostate cancer (relative risk (RR) = 1.1, 95% CI: 0.7–1.8, test for trends = 0.45) [132]. Quantification of micronutrients in the diet in a group of 6403 volunteers did not find a significant linear relation between phosphorus intake and concentration of serum prostate specific antigen, but there was a non-linear relationship due to an inflection point at a daily intake of 1151 mg [133]. A comparison of 27 oral squamous cell carcinoma patients and 86 controls reported an increased intake of phosphorus in the former (1761 mg daily) than in the latter (1431 mg daily) group (Mann–Whitney U test *p*-value = 0.003) [71].

Comparison of the upper and the lower quartiles in 516 cases of colorectal adenomas indicated a decreased risk of the insurgence of neoplasm (RR = 0.70, 95% CI: 0.54–0.90, test for trends *p*-value = 0.005) [134]. Such a trend was not observed in the 172 colorectal cancer-associated cases in the same study (RR = 0.73, 95% CI: 0.48–1.10, test for trends *p*-value = 0.11). There was a slightly non-significant higher risk of bladder cancer when comparing cases in the upper over the lower tertiles of daily phosphorus intake (OR = 1.82, 95% CI: 0.95–3.49, test for trends *p*-value = 0.06) [135]. When comparing cases in the upper over the lower quintiles of daily phosphorus intake, there was a non-significant higher risk of prostate cancer (OR = 1.20, 95% CI: 0.79–1.84, test for trends *p*-value = 0.39) [136]. There was an increased relative risk of prostate cancer when comparing patients in the upper over the lowest quintiles of daily phosphorus intake (RR = 1.12, 95% CI: 1.03–1.23, test for trends, *p*-value = 0.003) in a cohort of 19 147 patients [137].

## 5. Iron and Iodine

Iron, like copper, is fundamental for cellular biochemistry due to its oxidoreductase properties [138]. In particular, the link between cancer and iron is due to its involvement in the oxidative status of the cell [65]. The bulk of dietary iron is absorbed in the duodenum, whereas a large proportion of iron for human physiology is derived by recycling old erythrocytes [139]. The iron found in fruits, vegetables, nuts, grains, and the majority of meat is ferric (Fe^3+^) and not associated with the heme group, whereas approximately 40% of meat-derived iron is part of the metalloporphyrin complex. Ferric iron is converted to ferrous iron (Fe^2+^) by the duodenal cytochrome B reductase and transported by divalent metal transporter 1 (DMT-1) inside intestinal epithelial cells (enterocytes), whereas, an independent process internalizes the heme group, which contains ferrous iron [140]. Colonocytes are less efficient in heme uptake than enterocytes because they express less DMT-1; as a result, these cells normally have low iron content. The meat-rich Western diet has been associated with a daily intake of about 15 mg of iron (10% of it actually being absorbed by the enterocytes) and has increased colorectal carcinoma (CRC) risk [138,141]. Vitamin C increases enterocytes heme-iron intake while calcium decreases it [142,143,144]. Ferrous iron is trapped inside proteinaceous shells made of ferritin, forming molecular cages capable of holding up to 5000 iron atoms [145]. Iron is released into the bloodstream by the enterocytes via ferroportin, where it is converted to ferric form and bound to plasma transferrin [140]. The amount of serum iron is regulated by modulating the expression of ferroportin and hepcidin; the latter binds ferroportin causing the degradation of this membrane transporter [140]. Excess serum iron is deposited in several organs including liver, pancreas, and heart causing several diseases such as cirrhosis, cardiomyopathy, diabetes, and cancer. The oncogenesis of CRC and HCC has been linked to excess cellular iron and the process is the same in the two tissues [146]. In animal models, a diet high in red meat has been shown to increase the amount of heme in the colon, leading to an increase in the frequency and size of colorectal polyps [143,147,148]. Colonocytes will be used to describe the oncogenic process linked to iron.

The amount of iron inside cells is tightly regulated, and oncogenesis associated with this micronutrient is caused by high levels of a powerful oxidoreductive species in the cellular environment. Through the Fenton reaction (Fe^2+^ + H_2_O_2_ + H^+^ → Fe^3+^ + H_2_O + HO•), iron can produce hydroxyl radical (HO•) that belongs to ROS. Lipid peroxidation, in particular, will increase the number of radicals within the cell, alter cellular permeability, and initiate an inflammatory response [149]. Vitamin C, in the form of ascorbate (AscH^–^) rescues leaked ferrous iron by converting ferric iron to Fe^2+^, which can be taken up by ferritin, but in the presence of a large amount of iron, it boosts the Fenton reaction by providing even more ferrous iron: Fe^3+^ + AscH^−^ → Fe^2+^ + H^+^ + Asc•^−^ [150]. The ascorbic radical (Asc•^−^) can also affect the mitochondrial respiration producing a ROS burst that causes even further cellular damage [120]. ROS is also part of several signal pathways (such as NF-κB, PI3K, and MAPK), hence, high levels of these free radicals promote aberration in cellular proliferation and inflammation [151,152]. Iron is also included in the catalytic centers of the mitochondrial complexes I (nicotinamide adenine dinucleotide hydride (NADH) dehydrogenase), II (succinate dehydrogenase), and III (cytochrome bc-1 complex) [153].

It is known that the amount of cellular iron is directly related to the proliferation of the cells [65], due to the fact that iron is part of several enzymes that are involved in the DNA replication (polymerases α, ε, and δ, DNA primase, and helicase) and repair (polymerase ζ, helicase, DNA glycosylase, Fe^2+^/2-oxoglutarate-dependent dioxygenase) [154]. Iron is also a component of the ribonucleotide reductase (RNR) enzyme that transforms ribonucleotides to deoxyribonucleotides, without which DNA replication would be impossible [155,156]. Moreover, iron can promote cellular proliferation by activating the Wnt signaling pathway in presence of adenomatous polyposis coli (APC) mutations, thus, increasing the CRC risk [157].

Iodine is incorporated primarily in thyroid hormones, whereas its non-hormonal activity is less understood but is believed to be also involved in the oxidative state of the cells [158]. The biological role of iodine in oncogenesis is due to its role as a ROS scavenger and lipoperoxidation inhibitor in the reduced form of iodide (I^−^) [159]. The RDA for iodine is 150–299 μg daily, and abnormal intake is associated with widespread dysfunctions, including disrupting the immune system.

The risk of developing any cancer was lower in people under a regime of low iron intake (*n* = 60) than in people under regular diet (*n* = 38), with an HR of 0.65 (95% CI: 0.43–0.97, test for trends *p*-value = 0.36) [160]. Interestingly, a study on 11 026 cancer cases showed that the cancer risk increased at high (above 80 µg/dL) and low (below 60 µg/dL) serum iron [161]. Low serum iron was associated with an HR of 1.18 (95% CI: 1.08–1.29), whereas high levels had an HR of 1.25 (95% CI: 1.16–1.35).

A meta-analysis of the scientific literature reported that high serum iron was associated with a higher risk of colorectal cancer (RR = 1.02, 95% CI: 0.75–1.38) as was high intake of heme iron (RR = 0.93, 95% CI: 0.62–1.42) [162]. Other meta-analysis did not support such trends, indicating an RR of 0.97 (95% CI: 0.82–1.14) in relation to iron intake [163]. Such disparate findings highlight the paucity of knowledge about the role of iron in oncogenesis and the need for additional research.

Serum iron below 60 µg/dL was associated with more prolonged survival of gastric cancer patients in Stage III (log-rank test *p*-value = 0.033) [164]. A comparison of 27 oral squamous cell carcinoma patients and 86 controls reported an increased iron intake in the former (22.4 mg daily) than in the latter (18.9 µg daily) group (Mann–Whitney U test *p*-value = 0.029) [71]. The comparison of blood iron in oral squamous-cell carcinoma was significantly higher (*t*-test *p*-value < 0.001) than in healthy controls (194.6 and 128.6 µg/dL, respectively) [73].

A comparison of breast cancer patients (*n* = 24) and healthy volunteers (*n* = 48) showed a higher proportion of cases with urine iodine concentration above 200 µg/L (33.3%) compared to the controls (2.1%) (χ^2^ test *p*-value = 0.001) [165]. A study of 5926 breast cancer patients found an HR of 1.06 (95 % CI: 0.90–1.25) when comparing the upper quartile of serum iron to the first quartile, though no significant relationship was found [166]. Measurement of iodine concentration by computed tomography in 80 rectal cancer patients showed a correlation with the marker of cellular Ki-67 (Spearman correlation coefficient r = 0.344, *p*-value = 0.002) and the tumor biomarker hypoxia-inducible factor 1α (HIF-lα) (r = 0.598, *p*-value < 0.001), the latter used at a threshold of 0.584 g/cm^3^ to attain a 78% sensitivity and 87% specificity in the detection of cancer [167].

Spectroscopic analysis of brain sections reported a significantly lower iron concentration in tumoral masses than in surrounding healthy tissues (0.118 and 0.088 µg/cm^2^, respectively; Mann–Whitney U test *p*-value < 0.01) [75]. When patients in the upper quartile were compared to those in the lower quartile, low iron concentration in urine was associated with a lower risk of developing large thyroid tumors, with an OR of 0.56 (95% CI: 0.35–0.9, test for trends *p*-value = 0.026) [168].

## 6. Calcium

Calcium is part of many signaling pathways as second messenger and it takes part directly in the cell-to-cell adhesion, hence calcium impairment has a profound effect on the cell cycle, cellular proliferation, resistance to apoptosis, and metastasis [169,170]. Calcium is also involved in the folding of proteins synthesized in the endoplasmic reticulum (ER) [171]. In particular, calnexin (CNX) is an enzyme embedded in the ER’s membrane that can interact with nascent glycosylated proteins in a calcium-dependent manner [172].

Calcium is stored inside the ER by specific membrane pumps known as sarco/endoplasmic reticulum calcium ATPases (SERCA) and released in the cytoplasm upon stimulation with IP3 by channels known as IP3 receptors (IP3R) [173]. SERCA has been proposed as a tumor biomarker due to the relation between its altered expression and cancer development [174]. Calreticulin (CRT) is present in the ER’s lumen and regulates both the intake and release of calcium [175]. Both CNX and CRT has been linked to several diseases including cystic fibrosis, atherosclerosis, Alzeimer’s disease, and cancer [171] and both can interact with the zinc-containing endoplasmic reticulum resident protein 72 (ERp72) [176]. ERp72 over-expression is associated to cellular proliferation and has been identified not only as part of the CNX/CRT complex for the maturation of glycoprotein (where it acts as a disulfide isomerase), but is also involved in the rapid internalization and nuclear translocation of vitamin D3 (in the form of calcitriol), the activation of several transcription factors (such as NF-κB, mammalian target of rapamycin complex 1 (mTORC1), and eukaryotic translation initiation factor 4E binding protein 1 (4E-BP1)), and DNA repair (by modulating the activity of the redox factor-1 and by directly regulating the phosphorylation of histone H1AX) [177]. Moreover, calcium is loaded onto calmodulin (CaM), which binds and modulates not only one member of the Ras family of GTPases, Kirsten rat sarcoma (K-Ras), but many kinases, phosphatases, membrane pumps, transcription factors, and proteins of the extracellular matrix [178,179].

The tissue calcium levels were measured at 1431 mg/kg in healthy subjects (*n* = 49) and 657 mg/kg in prostate cancer patients (*n* = 50), with a statistically significant difference (*p*-values < 0.001) [103]. Serum calcium levels were similar between ovarian cancer patients and healthy controls (9.34 and 9.31 mg/dL, respectively) but the normalization by serum albumin content was significantly higher in cases than in controls (9.95 mg/dL and 9.53 mg/dL, respectively; *p*-value < 0.01) [180]. High serum calcium levels reduced the risk of breast cancer: the ratio of the fourth over the first quartiles in a group of 10 863 patients gave HR = 0.94 (CI: 0.8–0.99, test for trends *p*-value = 0.04) [181].

The comparison of blood calcium in oral squamous-cell carcinoma was significantly higher (*t*-test *p*-value < 0.001) than in healthy controls (14.7 and 9.4 mEq/L, respectively) [73]. Spectroscopic analysis of brain sections reported a significantly lower calcium concentration in tumoral masses than in surrounding healthy tissues (0.088 and 0.182 µg/cm^2^, respectively; Mann–Whitney U test *p*-value < 0.01) [75].

Calcium levels can also identify patients at higher risk for metastases. However, a cut-off of 2.5 mmol/L of calcium could identify bone metastases in bladder cancer patients with a sensitivity of 32% and a specificity of 94%, with high calcium levels providing an increased risk of metastasis (OR = 13.049, CI: 3.836–44.384, *p*-value < 0.001) [182]. A cut-off of 2.7 could identify HCC patients at risk of ocular metastasis with a sensitivity of 19% and a specificity of 97%, with high calcium levels providing an OR of 1.062 (CI: 1.028–1.096) for the risk of metastasis [183].

## 7. Discussion

Nutrition’s role in oncogenesis is increasingly being researched and supported by new scientific data. As a result, scientists are becoming more and more interested in determining the role of micronutrients in DNA stability, epigenetic regulation, immunological response, and in assessing their role as biomarkers.

Tumorigenesis is extremely complex and varies greatly between tumor entities due to the involvement of multiple molecular processes. The present analysis revealed some intriguing tendencies, even though there is no unified trend relating mineral intake and cancer risk. Specifically, higher levels of copper, iron, and iodine were linked to higher cancer risk, but zinc, selenium, calcium, and phosphorus had a more mixed relationship. A higher mineral intake was linked to an increased risk of oral cancer (iron, selenium, phosphorus, and zinc). Higher levels of zinc and copper, but lower levels of selenium, were more associated with liver cancer. Prostate cancer, on the other hand, was associated with higher phosphorus intake. The association between minerals and risk of cancer in observational and intervention studies is summarized in Table 2, Table 3 and Table 4, respectively.

Remarkably, the observation that cancer risk increases with both low and high serum iron levels [161] implies that maintaining physiological levels of micronutrients is critical to avoiding an imbalance of cellular biochemistry that can promote oncogenesis. A balanced diet can ensure the intake of the Goldilocks’ quantities of micronutrients, but the depletion of minerals and vitamins in foodstuff makes this increasingly difficult. Regularly checking the mineral levels can help identify whether the physiological range has been met or if the “hidden hunger” has developed increasing the cancer risk.

Given the interdependence of many micronutrients, predicting the role of a single mineral on the oncogenic pathway is cumbersome. For example, serum phosphorus is also linked to calcium and vitamin D and the cellular bioavailability of iron depends on that of vitamin C. Such complexities suggest that minerals may not be suitable cancer biomarkers individually, but a panel of said micronutrients, including vitamins, may provide a more complete picture. Because multiple variables must be considered, it is plausible to expect that micronutrient panels would be best analyzed using machine learning approaches, providing personalized indices to assess a potential increased cancer risk.

An imbalanced diet that is often involved in cancer development, is a clear and present danger, especially in industrialized countries. It is not only the high food intake that represents a problem. The vast majority of consumers are unaware of the actual composition of the food or beverages they consume, a situation exacerbated by the fact that food safety authorities do not require manufacturers to declare the actual amounts of micronutrients present in the aliments [185]. The trend in micronutrient depletion reported in the last decades, coupled with the increasing prevalence of the “hidden hunger”, suggests that health issues associated with the nutritional deficiency will become more prominent in the near future. According to the World Health Organization, two billion individuals worldwide, including those in wealthy countries, suffer from micronutrient deficiency that is largely clinically undetected [186]. The addition of micronutrients to food (fortification) has been purposely introduced to fight the spread of nutritional deficiencies [187]. However, the effectiveness of this precaution is still debated and does not consider its role on cancer prevention [188,189,190,191].

While most knowledge on the medical effect of minerals is associated with high intakes, the impact of mineral deprivation on oncogenesis is still not fully understood. The most recognized medical conditions associated with minerals deficiencies, such as MD, are congenital and include stillbirth, high infant mortality, and impaired development. The role of the mineral deficiency in adulthood and its involvement in oncogenesis is still unclear. 

The data gathered here highlight a direct association between high intake of micronutrients (namely copper, iron, and iodine) and cancer risk. The message of this review may point to a potential contradiction: while the average amount of micronutrients in food is decreasing, a higher cancer risk is associated with a higher amount of minerals. The solution to this paradox could be two-sided. On the one side, while most mineral concentrations in food are reduced, some minerals are augmented; this is particularly true for phosphorus and iron, particularly from heme. Mineral depletion, on the other side, may cause cells to adopt a transformed phenotype that abnormally increases the intracellular amount of micronutrients, for example, through the aberrant expression of surface importers. 

Assuring a well-balanced diet is a difficult task, especially in a food market dominated by advertisements for “junk food”. Rather than relying on labels to report the amounts of selected ingredients (a process hampered by manufacturers’ use of incomprehensible chemical names for key health-associated micronutrients), it is more feasible to embrace population-wide nutritional education. If children are educated on a balanced diet beginning in primary school in a few generations, consumers will be the advisors of their nutritional intake without relying too much on labels. Nutrition deficiency will be reduced in this context, as will the biochemical risks associated with micronutrient overload.

## 8. Conclusions

In conclusion, the present review confirms the potential of micronutrients as biomarkers, although the strong interdependence of micronutrient levels, could necessitate looking at a wide range of micronutrients to increase significance of the findings. There was a tendency of a direct link between copper, iron, iodine, phosphorus, and zinc levels and development of different cancer types, with the exception of colon cancer. Selenium serum levels were instead inversely related to cancer risk. However, more data are needed to assess the effectiveness of these biomarkers and if they are suitable only for specific types of cancer. Clustered micronutrient analyses could help the establishment of suitable micronutrient panels with diagnostic value to predict individual cancer risk and to facilitate predictions about the prognosis of particular types of cancer. Furthermore, the individual necessity of supplementation with food supplements, especially micronutrients, should always be critically questioned as long as there is no evident deficiency.

## Figures and Tables

**Table 1:** Recommended daily intakes and related serum levels of the minerals reported in the present review *.

Mineral	RDA/PRI (µg/Day)	EAR/AR (µg/Day)	UL (µg/Day)	Serum Levels	Source	References
Iron	8000	6000	45,000	30 µg/L ^†^	Meat, fish, cereals, beans, nuts.	[12,13,14]
Zinc	8000–11,000	9400	25,000	≥800 µg/L ^‡^	Meat, legumes, eggs, fish, grains.	[12,15,16]
Selenium	30–70	70	300	47–145 µg/L	Meat, fish.	[12,17]
Phosphorus	700,000	580,000	n.d.	0.8–1.5 × 10^3^ µmol/L	Meat, fish.	[12,18]
Calcium	1,000,000	750,000	2,500,000	2500 µmol/L	Milk, fish, legumes.	[12,14,19]
Copper	900	1600	5000	1200 µg/L	Milk, fish, eggs, vegetables.	[12,20]
Iodine	150	95	600	40–80 µg/L	Marine products, eggs, milk, iodized salt.	[12,21]

* Depicted data refers to male adults. AR: average requirement; EAR: estimated average requirement; n.d.: not determined by EFSA; PRI: population reference intake; RDA: recommended daily allowance; UL: tolerable upper intake level. ^†^ as ferritin. ^‡^ not reliable indicator for zinc status.

**Table 2:** Summary of the association between minerals and risk of cancer in observational studies.

Mineral	Organ	Sample	Association *	Measure ^†^	Reference
Zinc	Breast	Tissue	Direct	Qt	[70]
	Brain	Tissue	Inverse	Qt	[76]
		Intake	Inverse	Qt	[75]
	Mouth	Intake	None	Qt	[71]
		Serum	Inverse	Qt	[73]
	Liver	Serum	None	HR	[72]
		Serum	Direct	Qt	[78]
	Colon	Tissue	Inverse	OR	[29]
Copper	Liver	Serum	Direct	HR	[72]
		Serum	Direct	Qt	[78]
	Mouth	Serum	Direct	Qt	[73]
	Colon	Tissue	Direct	OR	[29]
	Brain	Tissue	Inverse	Qt	[76]
		Serum	Direct	Qt	[77]
		Intake	Inverse	Qt	[75]
	Pancreas	Serum	Direct	Qt	[74]
Selenium	Esophagus	Tissue	Direct	Qt	[107]
	Prostate	Tissue	None	Qt	[103]
		Serum	None ^║^	Qt	[104]
	Any	Serum	None	Qt	[98]
		Serum	Inverse	Qt	[99]
	Liver	Serum	Inverse	Qt	[105]
		Serum	Inverse	Qt	[108]
	Colon	Serum	None ^¶^	IR	[106]
	Pancreas	Serum	Inverse	OR	[74]
	Breast	Serum	Inverse	HR	[109]
		Serum	Inverse	HR	[110]
	Lung	Serum	None	Qt	[100]
	Lung	Serum	Direct	HR	[101]
	Kidney	Serum	Inverse	OR	[102]
	Mouth	Intake	Direct	Qt	[71]
Phosphorus	Brain	Intake	Direct	Qt	[75]
	Prostate	Intake	None	RR	[133]
		Intake	Direct	Cr	[133]
		Intake	Direct	OR	[137]
		Intake	None	OR	[136]
	Colon	Intake	Inverse ^#^	RR	[134]
	Bladder	Intake	None	OR	[135]
	Mouth	Intake	Direct	Qt	[71]
Calcium	Prostate	Tissue	Direct	Qt	[103]
	Brain	Intake	Direct	Qt	[75]
	Ovary	Serum	Direct	Qt	[180]
	Breast	Serum	Inverse	HR	[181]
	Mouth	Serum	Direct	Qt	[73]
Iron	Any	Serum	Direct/Inverse	HR	[161]
		Intake	Direct	HR	[160]
	Stomach	Tissue	Direct	Qt	[164]
	Brain	Intake	Direct	Qt	[75]
	Mouth	Intake	Direct	Qt	[71]
		Serum	Direct	Qt	[73]
	Breast	Serum	None	HR	[166]
	Thyroid	Urine	Direct	OR	[168]
Iodine	Breast	Urine	Direct	Qt	[165]
	Rectum	Tissue	Direct	Cr	[167]

* Direct: high micronutrient concentration is linked to an increased risk of cancer; inverse: low micronutrient concentration is linked to an increased risk of cancer; none: no significance observed. ^†^ Cr: correlation; Qt: comparison of levels between groups; HR: hazard ratio; IR: incidence rate ratio; OR: odds ratio; RR: relative risk. ^║^ Observed inverse trend by ethnic stratification. ^¶^ Observed inverse trend by sex stratification. ^#^ Observed in adenomas but not in carcinomas.

**Table 3:** Summary of the mineral levels in the studies retrieved for the present work.

Mineral	Cancer Entity	Sample	Case Group (Cancer) Mean Mineral Content Number of Patients	Control Group (Healthy) Mean Mineral Content Number of Patients	Reference
Zinc	Breast	Tissue ^†^	3.5–19.5 ppm (*n* = 26)	0.8–11.4 ppm (*n* = 26)	[70]
	Glioblastoma	Tissue	0.0403 µg/cm^2^ (*n* = 11)	0.0285 µg/cm^2^ (*n* = 11)	[75]
		Tissue ^†^	0.20 g/kg (*n* = 6)	0.27 g/kg (*n* = 6)	[76]
	Colon	Serum	96.4 µg/dL (*n* = 966)	97.1 µg/dL (*n* = 966)	[29]
Copper	Glioblastoma	Tissue	0.0090 µg/cm^2^ (*n* = 11)	0.0079 µg/cm^2^ (*n* = 11)	[75]
		Tissue ^†^	0.48 g/kg (*n* = 6)	1.26 g/kg (*n* = 6)	[76]
		Serum	27.5 µmol/L (*n* = 52) ^‡^	19.7 µmol/L (*n* = 52) ^‡^	[77]
	Colon	Serum	138.6 µg/dL (*n* = 966)	135.8 µg/dL (*n* = 966)	[29]
	Pancreas	Serum	1432 µg/L (*n* = 100)	1098 µg/L (*n* = 100)	[74]
Selenium	Any	Serum	58.8 µg/L *	84.8 µg/L (*n* = 966)	[99,106]
	Esophageal	Tissue ^†^	0.73 µg/g (*n* = 30) ^‡^	0.59 µg/g (*n* = 30) ^‡^	[107]
	Prostate	Tissue	191 µg/kg (*n* = 49)	168 µg/kg (*n* = 49)	[103]
		Serum	0.13 µg/g (*n* = 467)	0.14 µg/g (*n* = 936)	[104]
	Breast	Serum	90.5 ng/mL (*n* = 100)	91.3 ng/mL (*n* = 1186)	[109]
	Liver	Serum	67.47 µg/L (*n* = 187)	108.38 µg/L (*n* = 120)	[105]
	Colon	Serum	84.0 µg/L (*n* = 966)	85.6 µg/L (*n* = 966)	[106]
	Pancreas	Serum	60.0 µg/L (*n* = 100)	76.0 µg/L (*n* = 100)	[74]
	Lung	Serum	166.00 ng/g (*n* = 48)	144.74 ng/g (*n* = 39)	[100]
	Renal	Serum	161.7 µg/L (*n* = 401)	288.8 µg/L (*n* = 774)	[102]
Phosphorus	Glioblastoma	Tissue	1.71 µg/cm^2^ (*n* = 11)	3.01 µg/cm^2^ (*n* = 11)	[75]
Iron	Glioblastoma	Tissue	0.037 µg/cm^2^ (*n* = 11)	0.118 µg/cm^2^ (*n* = 11)	[75]
	Oral	Serum	194.6 µg/dL *	128.6 µg/dL *	[73]
Calcium	Prostate	Tissue	657 mg/kg (*n* = 50)	1431 mg/kg (*n* = 49)	[103]
	Oral	Serum	14.7 mEq/L *	9.4 mEq/L *	[73]
	Ovary	Serum	9.34 mg/dL (*n* = 170)	9.31 mg/dL (*n* = 344)	[180]

* No number of people per group reported. ^†^ Comparison between tumoral mass and healthy surrounding tissues. ^‡^ Estimated from article’s figures using *WebPlotDigitizer* v. 4.5 [184].

**Table 4:** Summary of the daily intake of minerals in the studies retrieved for the present work.

Mineral	Cancer Entity	Case Group (Cancer) Mean Mineral Intake Number of Patients	Control Group (Healthy) Mean Mineral Intake Number of Patients	Reference
Zinc	Oral	12,851 µg/day (*n* = 27)	11,788 µg/day (*n* = 86)	[71]
	Bladder	14.5 mg/day (*n* = 198)	14.7 mg/day (*n* = 377)	[135]
Copper	Bladder	2.5 mg/day (*n* = 198)	2.8 mg/day (*n* = 377)	[135]
Selenium	Oral	142.9 µg/day (*n* = 27)	166.7 µg/day (*n* = 86)	[71]
Phosphorus	Oral	1761 mg/day (*n* = 27)	1431 mg/day (*n* = 86)	[71]
	Bladder	1898.3 mg/day (*n* = 198)	1940.4 mg/day (*n* = 377)	[135]
Iron	Oral	22.4 mg/day (*n* = 27)	18.9 mg/day (*n* = 86)	[71]
	Bladder	21.3 mg/day (*n* = 198)	23.1 mg/day (*n* = 377)	[135]
Calcium	Bladder	1127.2 mg/day (*n* = 198)	1194.5 mg/day (*n* = 377)	[135]

## Abbreviations

4E-BP1	eukaryotic translation initiation factor 4E binding protein 1
A20	zinc finger protein A20
AKT	Ak strain transforming
APC	adenomatous polyposis coli
AR	average requirement
AscH^−^	ascorbate
ATP7A	ATPase copper transporter 7A
ATOX1	antioxidant-1
Braf	rapidly accelerated fibrosarcoma isoform B
CaM	calmodulin
CI	confidence interval
CNX	calnexin
Cr	correlation
CRC	colorectal carcinoma
CRT	calreticulin
CytC	cytochrome c
DMT-1	divalent metal transporter 1
DNA-pol	DNA polymerase
EAR	estimated average requirement
ER	endoplasmic reticulum
Erp72	endoplasmic reticulum resident protein 72
ERK	extracellular signal-regulated kinase
GPx	glutathione peroxidase
HCC	hepatocellular carcinoma
HIF-lα	hypoxia-inducible factor 1α
HR	hazard ratio
IκB	NF-κB inhibitor
IKK	IκB kinase
IP3	inositol 1,4,5-trisphosphate
IP3R	inositol trisphosphate receptor
K-Ras	Kirsten rat sarcoma
LOX	lysyl oxidase
MAPK	mitogen-activated protein kinase
MAPKK	mitogen-activated protein kinase kinase
MD	Menkes disease
MEK	MAPK/ERK kinase
MEMO1	mediator of cell motility 1
mTORC1	mammalian target of rapamycin complex 1
MURR1	mouse U2af1-rs1 region 1
NADH	nicotinamide adenine dinucleotide hydride
NF-κB	nuclear factor kappa-light-chain-enhancer of activated B cells
OR	odds ratio
OS	overall survival
PI3K	phosphoinositide 3-kinase
PRI	population reference intake
ppm	parts per million
Q	coenzyme Q
Qt	quantitative comparison of levels between groups
Raf	rapidly accelerated fibrosarcoma
Ras	rat sarcoma virus
RDA	recommended daily allowance
RNR	ribonucleotide reductase
ROS	reactive oxygen species
RR	relative risk
Sec	selenocysteine
SECIS	Sec insertion sequence
Sep15	selenoprotein of 15-kDa
SERCA	sarco/endoplasmic reticulum calcium ATPase
SOD	superoxide dismutase
SPARC	secreted protein acidic and rich in cysteine
TR1	thioredoxin reductase 1
UL	tolerable upper intake level
ULK	Unc-51-like autophagy activating kinase
Wnt	wingless/int-1
XIAP	X-linked inhibitor of apoptosis
